# Effects of asparaginase-associated pancreatitis in children with haematological tumours

**DOI:** 10.3389/fonc.2024.1472049

**Published:** 2024-10-08

**Authors:** Hui-jiao Tang, Chang-cheng Chen, Wen-ting Hu, Shu-hong Shen, Jing-qing Zeng, Sheng Ding, Zhao-hui Deng

**Affiliations:** ^1^ Department of Pediatric Gastroenterology, Shanghai Children’s Medical Center, School of Medicine, Shanghai Jiao Tong University, Shanghai, China; ^2^ Department of Pediatric Hematology and Oncology, Shanghai Children’s Medical Center, School of Medicine, Shanghai Jiao Tong University, Shanghai, China

**Keywords:** asparaginase, leukaemia, pancreatitis, event-free survival, complications

## Abstract

**Background:**

Asparaginase-associated pancreatitis (AAP) is a major challenge for continuing asparaginase therapy. We aimed to investigate the acute and long-term complications and survival rates related to first and second AAP episodes in Chinese children with haematological malignancies.

**Methods:**

We retrospectively analysed clinical data of children with pancreatitis who received asparaginase chemotherapy for acute lymphoblastic leukaemia (ALL), acute mixed cell leukaemia, and non-Hodgkin’s lymphoma at Shanghai Children’s Medical Center from November 2013 to November 2023.

**Results:**

Of the 76 children included in the study, 12 had local complications (15.79%), with no deaths recorded. Systemic complications manifested in 28 patients (36.84%), resulting in 3 deaths (3.95%). Four patients (5.26%) developed long-term complications (chronic pancreatitis or insulin-dependent diabetes mellitus). No significant differences in local or long-term complications were recorded between children in the asparaginase re-exposed (n=39) and non-re-exposed (n=45) groups. Among the re-exposed patients, eight (25.81%) experienced a second attack without fatalities or complications. Survival analysis of intermediate- to high-risk patients revealed a significantly higher event-free survival (EFS) rate for the re-exposed group than for the non-re-exposed group. The second AAP episode’s occurrence and severity had no relation to the first AAP episode’s severity, and the second AAP episode was significantly less severe than the first (*p*<0.001).

**Conclusions:**

The second AAP episode’s occurrence is unrelated to the first AAP episode’s severity, and the second AAP episode’s severity is significantly lower than that of the first. Further, asparaginase therapy could improve EFS in children with intermediate and high-risk ALL.

## 1 Introduction

Asparaginase is a basic chemotherapy component in paediatric acute lymphoblastic leukaemia (ALL) and lymphoma. It inhibits protein synthesis in tumour cells by breaking down asparagine in plasma into aspartic acid and ammonia, leading to cell death (1). Extensive clinical data and studies have demonstrated that asparaginase chemotherapy in children with leukaemia and lymphoma can improve remission and overall survival rates. However, asparaginase is associated with various toxicities, including hypersensitivity, pancreatitis, hyperlipidaemia, hepatotoxicity, thrombosis, and osteonecrosis, which may lead to the discontinuation of asparaginase therapy (2). This could potentially be associated with a decreased event-free survival rate in children, with asparaginase-associated pancreatitis (AAP) being the primary cause of discontinuation (3–6).

Previous studies have indicated that severe pancreatitis is the main reason for asparaginase interruption. Children with mild AAP are usually allowed to complete asparaginase treatment as scheduled. However, the incidence of severe pancreatitis in patients with AAP is 39.5%, which means that more than one-third of children with AAP may discontinue asparaginase. Some have argued that because the truncation of asparaginase is a key factor in the relapse of haematological malignancies in children, it is necessary to re-evaluate the risks of AAP and the criteria for re-exposure to asparaginase (7). The Ponte Di Legno Toxicity Working Group (PTWG) gathered comprehensive data on AAP in children with ALL from 18 countries (8). This large cohort study investigated the severity, associated complications, and risk factors of AAP for first episodes and second episodes after re-exposure to asparaginase. The risk of a second AAP episode after re-exposure to asparaginase was not related to the first AAP episode’s severity. More importantly, compared to the first AAP episode, the overall risk of complications and mortality from a second AAP event was not higher. Accordingly, asparaginase re-exposure should be determined mainly based on the anticipated requirement for asparaginase to achieve effective anti-leukaemia results.

We retrospectively analysed the acute and long-term complications and survival rates related to the first and second AAP episodes in Chinese children with haematological malignancies.

## 2 Materials and methods

### 2.1 Patient information

The Institutional Review Board of Shanghai Children’s Medical Center, which is affiliated with Shanghai Jiao Tong University School of Medicine, Shanghai, approved this study (SCMCIRB-YKW2021021). All the participants’ legal guardians provided written informed consent. Using the World Health Organization’s classification of myeloid neoplasms and acute leukaemia (9), a retrospective analysis was conducted on children treated with asparaginase for ALL, acute mixed-cell leukaemia, and non-Hodgkin lymphoma (NHL) at Shanghai Children’s Medical Center diagnosed with AAP between November 2013 and November 2023. The collected data included demographics, medication details, clinical manifestations related to pancreatitis, laboratory results, imaging findings, treatment, acute and long-term complications, re-exposure status, and prognosis. The median follow-up duration was 907.5 days. The inclusion criterion was children under 18 years of age diagnosed with acute pancreatitis following asparaginase administration. The exclusion criteria included pancreatitis from other causes, pre-existing abnormal pancreatic enzymes or imaging findings prior to asparaginase therapy, and the absence of relevant clinical data on AAP.

### 2.2 Diagnostic criteria and definitions

The standard diagnosis of AAP (according to the revised Atlanta criteria by the PTWG) requires that acute pancreatitis be diagnosed within 50 days after asparaginase therapy, with at least two of three specified criteria: (1) abdominal symptoms indicative of pancreatitis (acute, persistent, and severe upper abdominal pain); (2) serum amylase or lipase levels, or both, three times or more above the upper limit of normal (ULN); and (3) imaging findings characteristic of acute pancreatitis (8, 10).

The AAP severity was graded according to the PTWG guidelines: Grade 1, mild pancreatitis, with clinical manifestations and pancreatic enzymes at least three times the ULN for a maximum of 72 hours; Grade 2, severe pancreatitis, with clinical manifestations and/or pancreatic enzymes at least three times the ULN for more than 72 hours, or haemorrhagic pancreatitis, pancreatic abscess, or pseudocysts; and Grade 3, death from AAP (10).

Persistent complications owing to AAP were characterised by a long-term requirement for insulin therapy and recurring abdominal pain lasting for at least one year following diagnosis of asparaginase-associated complications, including insulin-dependent diabetes mellitus, chronic pancreatitis, and recurrent abdominal pain (8, 11). Local complications owing to AAP included pancreatic pseudocysts, peripancreatic fluid collections, acute necrotic collections, and walled-off necrosis (12).

Systemic complications owing to AAP included systemic inflammatory response syndrome (SIRS), sepsis, acute respiratory distress syndrome (ARDS), multiple organ dysfunction syndrome (MODS), intra-abdominal hypertension, and abdominal compartment syndrome (12–15).

### 2.3 Outcomes

The primary outcome was a comparison of acute and long-term complications of AAP in the first or second episode following re-exposure to asparaginase. Secondary outcomes included event-free survival (EFS) in all children and children in the intermediate-high risk group with AAP.

### 2.4 Statistical methods

Analyses were performed using SPSS 26.0 (IBM Corp., Armonk, NY, USA) and GraphPad Prism 9.0.0 (GraphPad Software, Boston, MA, USA) software. Based on the results of the Shapiro–Wilk test, continuous variables are expressed as means and standard deviations (x ± s) or medians and interquartile ranges (M [p25, p75]), and the comparison of normally and non-normally distributed variables of groups were performed using the t-test and Mann–Whitney U test, respectively. Categorical variables are presented as numbers and percentages and were analysed using the chi-squared or Fisher’s exact tests (when the expected cell number was less than 5). We utilised Kaplan–Meier curves to demonstrate survival probability and performed the log-rank test to compare survival differences between patient groups. To analyse the potential risk factors associated with EFS in children with AAP, we employed multivariate Cox regression models, presenting hazard ratios (HRs) and 95% confidence intervals (CIs). EFS was defined as the interval between AAP diagnosis and the date of the first or last follow-up event, with relapse, death, or a second malignancy considered competing events. Statistical significance was set at *p*<0.05.

## 3 Results

### 3.1 Patient characteristics

A total of 76 patients diagnosed with acute AAP (47 male and 29 female) were enrolled in the study. The median age at the time of AAP diagnosis was 7.84 (4.54, 12.42) years. Seventy-one patients were diagnosed with ALL after excluding 4 children who were diagnosed with AAP after recurrence of ALL, 49 in the intermediate-risk group, 13 in the low-risk group, and 5 in the high-risk group. Fifty-seven cases were of the B-cell type, and 14 were of the T-cell type. Thirteen cases had abnormal chromosome numbers, and 21 cases were positive for fusion genes, including *TEL-AML1* (n=8), *TCF3-PBX1* (n=2), *MLL-r* (n=2), and *Ph+/BCR-ABL* (n=9). One patient presented with acute mixed-cell leukaemia, and four had NHL. In the study population, 31 patients continued asparaginase therapy, and 45 discontinued it.

During the remission induction period, 42 children with ALL experienced AAP, whereas 8 developed AAP during the intensification period and 21 during the re-induction period. One child with MPAL developed AAP in induced remission after recurrence, 2 of the children with NHL developed AAP in induced remission and 2 in consolidation. Patients developed AAP after a median of 14 (8, 9) days from the last asparaginase dose, with a median of 2 (1, 5) administrations. Different asparaginase formulations were used in the patients: 64 patients received polyethylene glycol-conjugated asparaginase (PEG asparaginase), five received L-asparaginase, and seven were treated with Erwinase (Porton Biopharma, Salisbury, England) before the AAP event. Baseline characteristics of the study cohort are shown in **Table 1**.

**Table 1:** Baseline patient characteristics.

Characteristics	Number	%
Sex		
Male	47	61.84
Female	29	38.16
Primary diagnosis		
ALL	71	93.42
Mixed lineage leukaemia	1	1.32
NHL	4	5.26
Risk group		
LR	13	19.40
IR	49	73.13
HR	5	7.46
Immunophenotype		
B-cell	57	80.28
T-cell	14	19.72
Chromosomal aberration	21	27.63
Fusion gene detected	13	17.11
Asp re-exposure		
Yes	31	40.79
No	45	59.21
Stage of chemotherapy at AAP diagnosis		
remission induction	42	59.15
intensification	8	11.27
reinduction	21	29.58
Drug		
Peg-asp	64	84.21
L-Asp	5	6.58
Erwinase	7	9.21
Characteristics	M	P25, P75
Age	7.84	4.54, 12.42
BMI	15.56	14.22,17.86
Days from last Asp to diagnosis of AAP	14	8, 19
Times of Asp before diagnosis of AAP	2	1, 5
Follow-up time	794	145, 1176

ALL, acute lymphoblastic leukaemia; NHL, non-Hodgkin lymphoma; LR, low risk; IR, intermediate risk; HR, high risk; Peg-asp, polyethylene glycol-conjugated asparaginase; L-Asp, L-Asparaginase; Erwinase, Erwinia asparaginase; Asp, asparaginase; BMI, Body Mass Index.

### 3.2 Impact of the first AAP episode

According to the PTWG pancreatitis grading, 18 cases (23.68%) were Grade 1, 55 (72.37%) were Grade 2, and 3 (3.95%) died from AAP (classified as Grade 3). Twelve patients (15.79%) experienced local complications, including nine cases of pancreatic pseudocysts and three cases of peripancreatic fluid collections. Pseudocysts and peripancreatic fluid collections resolved after a median of 131 and 7 days, respectively, with one case of a pseudocyst progressing to chronic pancreatitis. Systemic complications occurred in 28 patients (36.84%), including SIRS in 21, sepsis in 17, ARDS in 6, and MODS in 23, resulting in 3 deaths. Long-term complications were observed in four patients (5.26%), including three with chronic pancreatitis (3.95%) and one with insulin-dependent diabetes mellitus (1.32%). After endoscopic retrograde cholangiopancreatography (ERCP), the three cases of chronic pancreatitis did not recur, and the child with diabetes mellitus discontinued insulin therapy after 392 days of follow-up (**Table 2**).

**Table 2:** Impact of the first asparaginase-associated pancreatitis episode.

Characteristics	Number	%
AAP severity		
Mild	18	23.68
Severe	55	72.37
Death	3	3.95
Local complications		
Pancreatic pseudocyst	9	11.84
Peripancreatic fluid collection	3	3.95
Systemic complications		
Systemic inflammatory response syndrome	21	27.63
Sepsis	17	22.37
Acute respiratory distress syndrome	6	7.89
Multiple organ dysfunction syndrome	23	30.26
Long-term complications		
Chronic pancreatitis	3	3.95
Insulin-dependent diabetes mellitus	1	1.32

AAP, asparaginase-associated pancreatitis.

### 3.3 Re-exposure to asparaginase after AAP

Based on re-exposure to asparaginase after the first episode of AAP, the 84 patients with AAP were divided into re-exposure group (n=39) and non-re-exposure group (n=45). There were no significant differences between the two groups in terms of the number of days of abdominal pain (*p*=0.113), the interval between the onset and normalisation of serum amylase levels (*p*=0.749), systemic complications (*p*=0.063), and long-term complications (*p*=0.448). However, the highest serum amylase levels (*p*=0.037) and the incidence of local complications (*p*=0.026) in the re-exposure group were significantly lower than those in the non-re-exposure group (**Table 3**).

**Table 3:** Comparison of patients who continued and discontinued asparaginase.

Characteristics	Total	Re-exposure group	No re-exposure group	Statistic	*p*-value
Number of patients, n (%)	84 (100%)	39 (40.79%)	45 (59.21%)	/	/
Days of abdominal pain	4.00 (1.63, 8.00)	4.00 (1.00, 7.00)	6.00 (3.00, 10.00)	Z=-1.59	0.113
Peak value of AMS at diagnosis of AAP	429.50 (232.00, 743.25)	333.00 (190.00, 655.00)	530.00 (261.00, 911.00)	Z=-2.08	0.037*
Days from AMS elevation to normalization	7.50 (4.75, 14.25)	7.00 (4.00, 14.00)	8.00 (5.00, 15.00)	Z=-0.32	0.749
**Local complications**				χ²=4.99	0.026*
Yes	12 (14.29)	2 (5.13)	10 (22.22)		
No	72 (85.71)	37 (94.87)	35 (77.78)		
**Systemic complications**				χ²=3.45	0.063
Yes	28 (33.33)	9 (23.08)	19 (42.22)		
No	56 (66.67)	30 (76.92)	26 (57.78)		
**Long-term complications**				χ²=0.58	0.448
Yes	5 (5.95)	1 (2.56)	4 (8.89)		
No	79 (94.05)	38 (97.44)	41 (91.11)		
Chronic pancreatitis	3 (3.57)	0 (0.00)	3 (6.67)	χ²=1.11	0.293

AMS, amylase; AAP, asparaginase-associated pancreatitis.

*Statistically significant.

Among the 31 children who were re-exposed to asparaginase after AAP, eight (25.81%) experienced a second attack, including seven cases of Grade 1 and one case of Grade 2 AAP, with no deaths reported. There were no local, systemic or long-term complications in the re-exposed children.

There was a significant difference in the severity of the first AAP episode compared to that of the second (*p*<0.001), with the first being more severe. There was no significant correlation between the first AAP episode’s severity and the occurrence of a second episode (*p*=1.000), and there were no significant differences in the occurrence of local (*p*=0.434) or systemic complications (*p*=0.090) or survival outcomes (*p*=0.348) between the first and second AAP episodes (**Table 4**). In the same patient, the severity of the second AAP episode was not significantly related to the severity of the first (*p*=0.063), and there was no significant correlation in terms of systemic complications (*p*=0.125).

**Table 4:** Comparison of the first and second asparaginase-associated pancreatitis episodes.

Variable	The first AAP (n=68)	The second AAP (n=8)	Statistic	*p*-value
**AAP severity**			Z=3.58	<0.001*
Mild	18 (26.47%)	7 (87.50%)		
Severe	44 (64.71%)	1 (12.50%)		
Dead	6 (8.82%)	0 (0.00%)		
Local complication	12 (17.65%)	0 (0.00%)	χ²=0.61	0.434
Systemic complication	27 (39.71%)	0 (0.00%)	χ²=2.88	0.090
**Primary disease outcome**			χ²=0.88	0.348
Alive	54 (79.41%)	8 (100.00%)		
Death	14 (20.59%)	0 (0.00%)		

AAP, asparaginase-associated pancreatitis.

*Statistically significant.

### 3.4 Survival analysis

**Figure 1:**
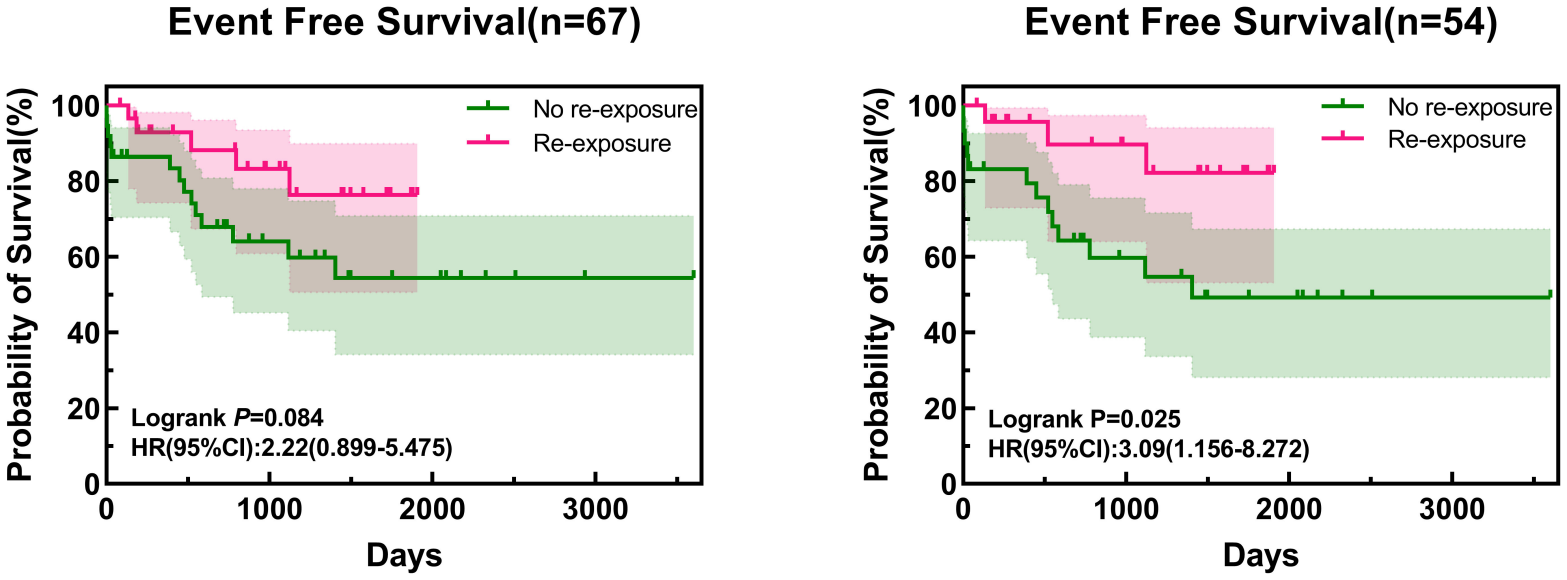
Survival curve of all patients and intermediate- to high-risk patients. HR, hazard ratio; CI, confidence interval.

**Figure 2:**
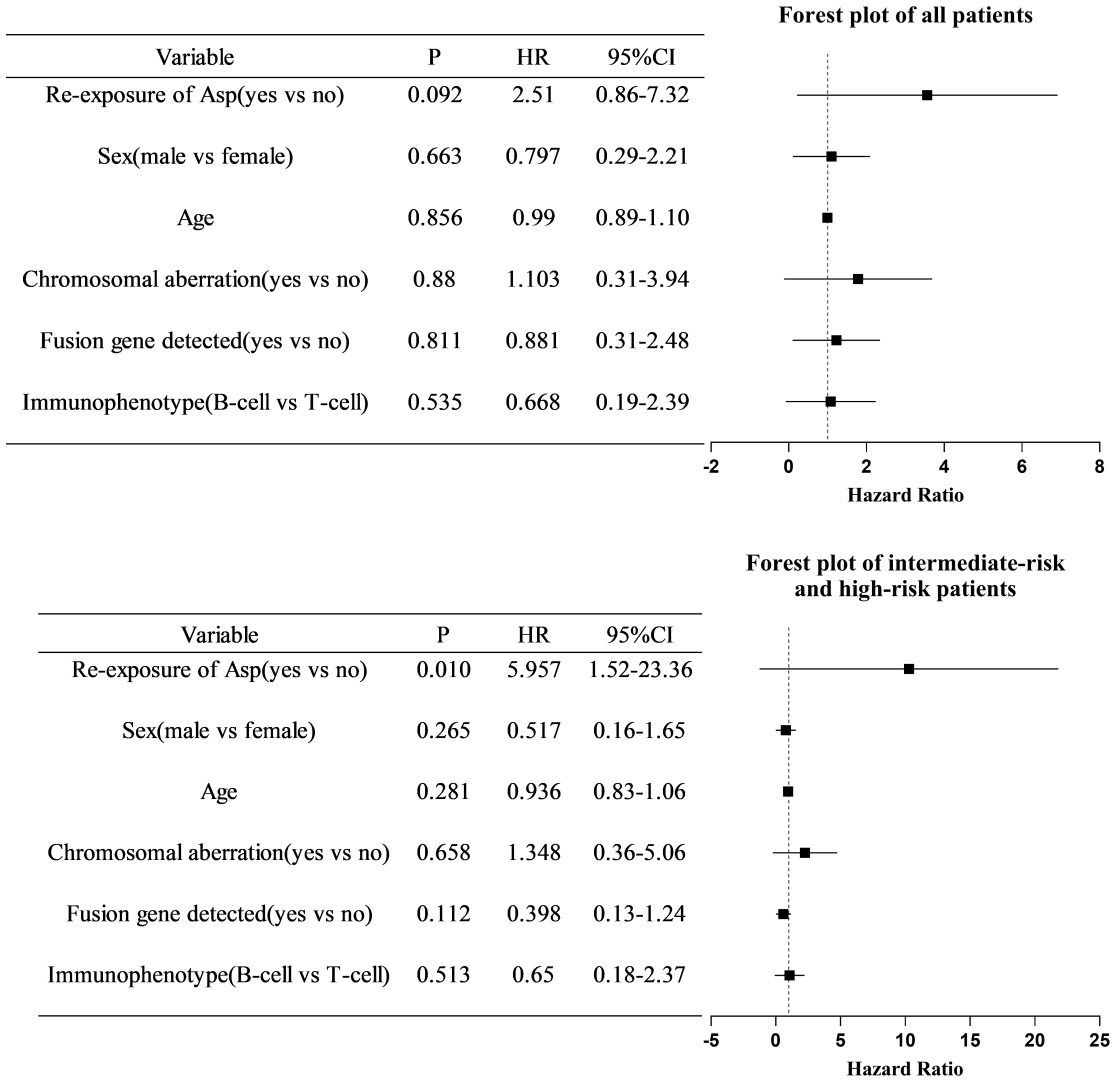
Multivariate COX regression analysis of risk factors for event-free survival in all patients and intermediate- to high-risk patients. COX, cyclooxygenase; HR, hazards ratio; CI, confidence interval; Asp, asparaginase.

After excluding five children with non-ALL and four children diagnosed with AAP after relapse of ALL, the EFS of all enrolled children who were re-exposed to asparaginase was not significantly higher than that in children who were not re-exposed (76.4% vs. 54.4%, respectively; log-rank *p*=0.084) (**Figure 1**). Multivariate Cox regression analysis, after adjusting for age, sex, immunotyping and presence of chromosomal abnormalities and fusion genes, showed that asparaginase re-exposure had no significant effect on the EFS of children (*p*=0.092; HR: 2.510; 95% CI: 0.861-7.315) (**Figure 2**). However, survival analysis of intermediate-risk and high-risk patients revealed that the EFS of children in the asparaginase re-exposure group was increased compared to that of children in the non-re-exposure group (82.2% vs 49.3%, respectively; log-rank *p*=0.025) (**Figure 1**). In the multivariate Cox regression analysis, after adjusting for age, sex immunotyping, and the presence of chromosomal abnormalities and fusion genes, the presence of asparaginase re-exposure was an independent risk factor for EFS in children with intermediate-high risk ALL (*p*=0.010; HR: 5.957; 95% CI: 1.519–23.361) (**Figure 2**).

## 4 Discussion

Acute and long-term complications in children with AAP are a primary concern for clinicians, serving as a key indicator for identifying patients who should have restricted exposure to asparaginase. This study assessed acute and long-term complications of first and second AAP episodes in children with haematological tumours, and it is the largest single-centre study on AAP in children in China.

Few studies have examined the complications of AAP in children. Kearney et al. (16) reported that of 403 patients with ALL, 28 (7%) experienced AAP, with five (18%) developing pseudocysts. None of the patients progressed to chronic pancreatitis, long-term exocrine pancreatic insufficiency, or required long-term insulin therapy. Among the 28 patients with AAP, 16 (57%) were re-exposed to asparaginase, and 10 experienced a second episode of AAP, one of which resulted in a pseudocyst, whereas the others recovered spontaneously, with no deaths from AAP and no decline in EFS. Mauney et al. (17) documented 34 patients with AAP, with 7 (20.6%) requiring admission to the intensive care unit, 17 (50%) having organ failure or pancreatic necrosis, 23 (67.6%) developing ascites, 10 (29.4%) having peripancreatic fluid, and 18 (52.9%) having pleural effusions. Despite severe complications following AAP, there were no deaths directly attributed to AAP. Our study found that the incidence of local complications was 15.79%, including nine instances of pancreatic pseudocysts and three of peripancreatic fluid, which subsequently resolved. Except for one child with a pseudocyst that progressed to chronic pancreatitis, none of the other children developed long-term complications or died owing to local issues. The systemic complication incidence rate was 36.84%, including 21 cases of SIRS, 17 of sepsis, 6 of ARDS, and 23 of MODS, resulting in three deaths. Long-term complications were observed in 5.26% of the patients, including three with chronic pancreatitis and one with insulin-dependent diabetes mellitus. Treatment with ERCP resulted in no recurrence in the three cases with chronic pancreatitis. The proportion of severe AAP cases in this study was higher than that in other studies, which may be related to the difference in the definition of AAP grade and chemotherapy regimen. Acute complications and long-term complications had little impact on the children’s quality of life. Deaths from AAP were primarily owing to systemic complications but did not exceed those caused by other forms of pancreatitis (2.0%–11.1%) (18).

Therefore, attention should be paid to systemic complications of AAP. Similarly, the incidences of chronic pancreatitis and insulin-dependent diabetes mellitus were relatively low, with minimal effect on the children’s quality of life. In our study, 31 children with AAP were exposed to asparaginase again, resulting in eight patients experiencing a second attack. The incidence of local and systemic complications in the second episode of AAP was not higher than in the first episode, and the recurrence of AAP was unrelated to the severity of the first episode of AAP. Even in the same child, the incidence of complications with recurrent AAP did not increase compared to the first episode, which aligns with the findings from Wolthers et al. (8).

Previous research has suggested that re-exposure to asparaginase should be considered only in children with mild AAP, which is defined as pancreatic enzymes exceeding three times the UNL and clinical symptoms lasting less than 72 hours, and absence of imaging evidence of pancreatic pseudocysts or necrosis (10, 19). However, Raja et al. (20) reported 45 AAP cases, 12 of which occurred after re-exposure to asparaginase, including three severe AAP cases at the first exposure. They found that further asparaginase therapy did not trigger pancreatitis recurrence, suggesting a broader consideration of the criteria for re-exposure to asparaginase after the first AAP episode. Similarly, in our study, 22 patients with severe AAP were re-exposed to asparaginase, and no complications or deaths occurred. We also believe that withdrawal should be done with caution so as not to affect the survival of patients with ALL.

Multiple studies have provided evidence that the truncation of asparaginase raises the haematological cancer relapse likelihood. In an international study by Rank et al. (21), which included 2,448 patients with ALL aged 1.0–45.9 years (including 168 patients with AAP), there was no significant increase in the 5-year cumulative recurrence rate among non-re-exposed patients with AAP compared to re-exposed patients with AAP, and there was no difference in the recurrence risk between patients with and those without AAP. Neither AAP nor asparaginase truncation owing to AAP was considered associated with an increased recurrence risk. Gupta et al. (22) enrolled 5,195 patients treated under the AALL0331 protocol and 3,001 patients treated under the AALL0232 protocol (aged 1–30.99 years) and found that in high-risk patients with ALL, asparaginase discontinuation was associated with worse disease-free survival (DFS), whereas it did not affect DFS in low-risk patients. Our study compared the EFS of children with AAP who were re-exposed to asparaginase with those who were not. The results showed that, regardless of risk stratification, asparaginase truncation did not increase tumour relapse or death risk in children with ALL. However, for children with ALL in the intermediate-high risk group, the EFS of children with AAP with interrupted asparaginase therapy was significantly lower than that of children who were re-exposed, with a 5.96-fold increased risk of tumour relapse or death. This may be owed to the demand for the intensity of asparaginase treatment in children with ALL in the intermediate and high-risk groups such that the use of a full course of asparaginase has an important impact on the prognosis of these patients, and insufficient exposure to asparaginase will lead to an increased risk of relapse. Therefore, from the perspective of the impact of asparaginase withdrawal on haematological tumour relapse, for children with intermediate and high-risk ALL, the potential dangers of being re-exposed to asparaginase must be carefully weighed against the need for asparaginase to ensure survival.

The study has certain limitations. The retrospective nature and lack of systematic AAP screening present, potential for selection biases in favour of patients with more noticeable symptoms. AAP can easily be confused with sepsis if pancreatic enzyme levels are not measured. The study involved a limited number of participants and had insufficient follow-up time for certain children. Survival analysis was not possible owing to the limited number of patients with ALL in the low-risk group who had confirmed AAP. The conclusions drawn require further validation through multicentre studies with larger sample sizes.

In conclusion, this study demonstrated that (1) in ALL treatment, identifying AAP early is essential to preventing progression to systemic complications and reducing mortality; (2) active treatment and multidisciplinary cooperation can effectively mitigate the harm caused by severe AAP; (3) the occurrence of a second AAP episode is not related to the severity of the first, and the severity of a second AAP episode is significantly lower than that of the first; and (4) further asparaginase therapy could improve the EFS of children with intermediate and high-risk ALL. Given the significance of asparaginase in treating paediatric haematological malignancies, the decision to reintroduce asparaginase should be mainly based on its effectiveness in combating leukaemia, even in children with severe AAP.

## Data Availability

The original contributions presented in the study are included in the article/supplementary material. Further inquiries can be directed to the corresponding author.
